# Identification and validation of IgG N-glycosylation biomarkers of esophageal carcinoma

**DOI:** 10.3389/fimmu.2023.981861

**Published:** 2023-03-14

**Authors:** Huiying Pan, Zhiyuan Wu, Haiping Zhang, Jie Zhang, Yue Liu, Zhiwei Li, Wei Feng, Guiqi Wang, Yong Liu, Deli Zhao, Zhiyi Zhang, Yuqin Liu, Zhe Zhang, Xiangtong Liu, Lixin Tao, Yanxia Luo, Xiaonan Wang, Xinghua Yang, Feng Zhang, Xia Li, Xiuhua Guo

**Affiliations:** ^1^ Department of Epidemiology and Health Statistics, School of Public Health, Capital Medical University, Beijing, China; ^2^ Beijing Municipal Key Laboratory of Clinical Epidemiology, Capital Medical University, Beijing, China; ^3^ Centre for Precision Health, School of Medical and Health Sciences, Edith Cowan University, Joondalup, WA, Australia; ^4^ Department of Endoscopy, National Cancer Center/National Clinical Research Center for Cancer/Cancer Hospital, Chinese Academy of Medical Sciences and Peking Union Medical College, Beijing, China; ^5^ Cancer Centre, The Feicheng People’s Hospital, Feicheng, Shandong, China; ^6^ Department of Gastroenterology, Gansu Wuwei Cancer Hospital, Wuwei, Gansu, China; ^7^ Cancer Epidemiology Research Centre, Gansu Province Cancer Hospital, Lanzhou, Gansu, China; ^8^ Department of Occupational Health, Wuwei Center for Disease Prevention and Control, Wuwei, Gansu, China; ^9^ Department of Mathematics and Statistics, La Trobe University, Melbourne, VIC, Australia

**Keywords:** glycomics, esophageal squamous cell carcinoma, immunoglobulin G, glycosylation, biomarkers

## Abstract

**Introduction:**

Altered Immunoglobulin G (IgG) N-glycosylation is associated with aging, inflammation, and diseases status, while its effect on esophageal squamous cell carcinoma (ESCC) remains unknown. As far as we know, this is the first study to explore and validate the association of IgG N-glycosylation and the carcinogenesis progression of ESCC, providing innovative biomarkers for the predictive identification and targeted prevention of ESCC.

**Methods:**

In total, 496 individuals of ESCC (n=114), precancerosis (n=187) and controls (n=195) from the discovery population (n=348) and validation population (n=148) were recruited in the study. IgG N-glycosylation profile was analyzed and an ESCC-related glycan score was composed by a stepwise ordinal logistic model in the discovery population. The receiver operating characteristic (ROC) curve with the bootstrapping procedure was used to assess the performance of the glycan score.

**Results:**

In the discovery population, the adjusted OR of GP20 (digalactosylated monosialylated biantennary with core and antennary fucose), IGP33 (the ratio of all fucosylated monosyalilated and disialylated structures), IGP44 (the proportion of high mannose glycan structures in total neutral IgG glycans), IGP58 (the percentage of all fucosylated structures in total neutral IgG glycans), IGP75 (the incidence of bisecting GlcNAc in all fucosylated digalactosylated structures in total neutral IgG glycans), and the glycan score are 4.03 (95% CI: 3.03-5.36, P<0.001), 0.69 (95% CI: 0.55-0.87, P<0.001), 0.56 (95% CI: 0.45-0.69, P<0.001), 0.52 (95% CI: 0.41-0.65, P<0.001), 7.17 (95% CI: 4.77-10.79, P<0.001), and 2.86 (95% CI: 2.33-3.53, P<0.001), respectively. Individuals in the highest tertile of the glycan score own an increased risk (OR: 11.41), compared with those in the lowest. The average multi-class AUC are 0.822 (95% CI: 0.786-0.849). Findings are verified in the validation population, with an average AUC of 0.807 (95% CI: 0.758-0.864).

**Discussion:**

Our study demonstrated that IgG N-glycans and the proposed glycan score appear to be promising predictive markers for ESCC, contributing to the early prevention of esophageal cancer. From the perspective of biological mechanism, IgG fucosylation and mannosylation might involve in the carcinogenesis progression of ESCC, and provide potential therapeutic targets for personalized interventions of cancer progression.

## Introduction

Esophageal cancer (EC) is the seventh most common cancer type worldwide and ranks sixth in the cause of cancer-related death (1). In China, there have been an amount of estimated 0.25 million new cases of esophageal cancer and 0.19 million related deaths as of 2018, accounting for 43% and 37% of the global morbidity and mortality (2). The 5-year relative survival rate of the localized esophageal cancer at the point of confirmed diagnosis is 47%, while the rate declines to only 20% for all esophageal cancer patients (3). In addition, esophageal squamous cell carcinoma (ESCC) predominates sub-type of esophageal cancer and is among the most aggressive forms of squamous cell carcinoma. ESCC belongs to the most deadly malignancy with late stage diagnosis, metastasis, therapy resistance and frequent recurrence (4).

Most patients of ESCC lack obvious symptoms at the early stage and progress insidiously to a relatively advanced stage when detected (5). Therefore, exploring the reliable biomarkers associated with early stage of ESCC is critical for improving the prognosis and life quality of patients, which fits with in the paradigm of predictive medicine. Esophagogastroduodenoscopy (EGD) is the main method for screening EC in the clinical practice, and it is of high cost, discomfortable and invasive. In addition, there are some serum biomarkers recommended for the assistant screening of EC, such as carcinoembryonic antigen (CEA), P53-Ab, Cytokeration fragment antigen21-1 (CYFRA21-1), squamous cell carcinoma antigen (SCC), protein kinase D1 (PRKD1), matrix metalloproteinase 2 (MMP-2), tissue inhibitor of metalloproteinases-2 (TIMP-2) and serum macrophage colony-stimulating factor (M-CSF). However, these tumor markers could alter in various tumor types, and even relate with the acute infection (6–8). Therefore, it is of great significance to identify novel biomarkers of high specificity and sensitivity for the early detection of ESCC, contributing to the early diagnosis and prevention of ESCC.

The glycomics analysis is a promising ‘omics’ technology (9), providing novel biomarkers for diseases diagnosis and prognosis, which could advance the personalized medicine and intervention strategy (10). Immunoglobulin G (IgG), as the most abundant immunoglobulin in blood, constitutes approximately 75% of the serum immunoglobulin proteins (11). IgG activates a series of effector pathways, such as complement-dependent cytotoxicity (CDC), antibody-dependent cellular cytotoxicity (ADCC) and antibody-dependent cellular phagocytosis (ADCP) (12, 13), which are regulated by the N-linked glycosylation process at the Fc segment of IgG. N-glycosylation is one of the most common post-translational modifications of membrane and secretory proteins, with an important role in the biological processes, such as intercellular recognition, adhesion, communication and mutual interactions (14, 15). It plays an important role in the antibody functions and almost all the tumor markers approved by FDA are modified through glycosylation (16). The attached N-glycans on IgG are essential for the proper functional activity of the immune system. IgG N-glycosylation has been reported to be affected by the pathophysiological conditions, and thus associated with various diseases, such as the metabolic diseases (17–22), aging (23, 24), inflammatory and autoimmune diseases (25, 26) The profile of IgG N-glycans could alter its effector functions on tumor cells, and the variability of IgG N-glycosylation has also been identified in some tumor types (27–30).

Our previous study found that the IgG N-glycosylation profiles were independently associated with the esophageal precancerosis for squamous cell carcinoma beyond inflammation (31). However, the association of IgG N-glycosylation pattern with ESCC remains unknown to date. In this study, we investigated the variation of IgG N-glycans in the stages of normal, precancerosis and early ESCC. We aimed to develop a predictive score using IgG N-glycans data to improve the risk stratification and management of ESCC.

## Materials and methods

### Study design and population

**Figure 1:**
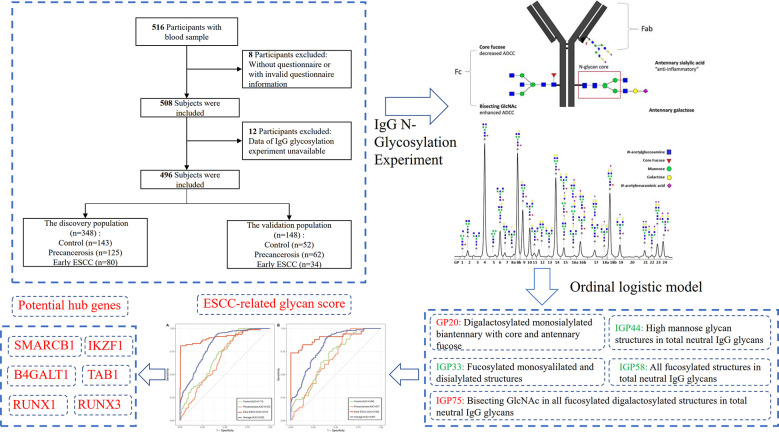
A schematic diagram of the study participants.

In total, 516 subjects voluntarily participated in this study and 496 individuals were finally recruited in the analysis according to the inclusion and exclusion criteria as shown in **Figure 1**. In 2018, 80 cases of early ESCC, 125 cases of precancerosis and 143 controls were enrolled from Feicheng People’s Hospital (Feicheng City, Shandong Province). Meanwhile, data of 34 early ESCC patients, 62 precancerosis patients and 52 controls were collected as validation group from Gansu Wuwei Tumor Hospital (Wuwei City, Gansu Province). This two-center respective case control study umbrellaed under a national screening project, aiming at the early screening and diagnosis of ESCC and other gastrointestinal cancers as described previously (31). Before the endoscopic screening, the demographic information, dietary habit, lifestyle, history of gastrointestinal disease and family history of gastrointestinal cancer were surveyed through a standardized questionnaire (**Supplementary Table S1**). The blood samples were collected and stored at -80°C for the subsequent experiment.

The following were the inclusion criteria: (1) providing informed consent prior to enrollment; (2) initial confirmed diagnosis of precancerosis or ESCC, or neither; (3) the required information and data of IgG glycosylation profile eligible. The exclusion criteria were as follows: (1) diagnosis of other gastrointestinal cancer (gastric cancer or intestinal cancer) before or at the screening; (2) history of mental illness, infectious disease, autoimmune diseases or and other malignant cancers; (3) women in pregnancy or lactation; (4) post-operation or post-radiochemotherapy.

The study was approved by the independent ethics committee of National Cancer Center/Cancer Hospital, Chinese Academy of Medical Sciences and Peking Union Medical College (grant number: 17–124/1380) and ethics committee of Capital Medical University (grant number: Z2019SY012). All participants provided their written informed consents before taking part in this study.

### Outcome definition

The diagnosis of precancerous esophageal lesions and early ESCC was according to the endoscopic screening and biopsy examination, while the judgment of the controls was only based on the endoscopic diagnosis. In a previous article we described the procedures of routine endoscopy examination (31). The controls in this study were defined as oesophagitis or normal, while esophageal precancerosis were defined as mild or moderate atypical hyperplasia, and the early ESCC included severe atypical hyperplasia, mucosal and submucosal carcinomas.

### Covariates

The body mass index (BMI) was defined as weight (in kilograms)/height^2^ (in meters squared) and the participants were grouped into <24 kg/m^2^ and ≥24 kg/m^2^. Systolic blood pressure (SBP) and diastolic blood pressure (DBP) were measured twice on the right arm using a standard mercury sphygmomanometer in a sitting position after the subjects had rested at least 10 minutes, and the mean value was used for the present analysis. Hypertension was defined as a self-reported history of hypertension, a mean SBP ≥140 mmHg or DBP ≥90 mmHg or taking antihypertensive medications. Education level was classified into illiteracy, primary school, middle or high school, bachelor degree or above. Marriage status was defined as married status or others. Family income was divided into less than and more than 50,000 yuan per capita per year. Smoke was defined as at least one cigarette per day in the past year, while drink was defined as at least 100 ml consumption of alcohol (content ≥50%) per day in the past year. Dietary frequency of pickled food, fried food, hot food and mildew food were grouped into never, seldom and often. History of gastrointestinal disease involved gastroenteritis and peptic ulcer. Family history of gastrointestinal cancer included esophageal cancer, gastric cancer and intestinal cancer.

### IgG N-glycosylation experiment

The glycosylation experiment and analysis involved four key processes: IgG isolation and purification from plasma, glycans enzyme digestion and release, fluorescence labeling and quantitative detection, as described previously (32, 33). In brief, IgG was isolated in a high-throughput manner, using 96-well protein G monolithic plates (BIA Separations, Slovenia), starting from 100 μl of plasma. Plasma was diluted 7× with phosphate buffered saline (PBS), applied to the protein G plate and washed. IgG was eluted with 1 ml of 0.1 M formic acid and immediately neutralized with 1 M ammonium bicarbonate. Then, the N-linked glycans were released by incubating at 37°C for 18-20 hours with 1.5 units of PNGase F. The released glycans were fluorescent labeled using 2-aminobenzamide at 65°C for 3 hours. After incubation samples were brought to 96% of acetonitrile (ACN) by adding 700 μl of 100% ACN and applied to each well of a 0.2 μm GHP filter plate. Solvent was removed by application of vacuum using a vacuum manifold. Loaded samples were subsequently washed 5× with 96% ACN. Fluorescently labelled N-glycans were separated by hydrophilic interaction chromatography on Acquity UPLC H-Class instrument (Waters, USA). Labelled N-glycans were separated on a Waters BEH Glycan chromatography column at 60°C, with 100 mM ammonium formate, pH 4.4, as solvent A and ACN as solvent B. Separation method used linear gradient of 75–62% acetonitrile at flow rate of 0.4 ml/min in a 27-min analytical run. Detect N-glycan fluorescence at excitation and emission wave lengths of 330 nm and 420 nm, respectively.

Finally, 24 direct glycan peaks (GPs) were quantitatively expressed with the percentage of the total integrated peak area, as presented in **Supplementary Figure S1**. In addition, 54 derived traits (IGPs) were derived to reflect the relative abundance of the specific structure, such as galactosylation, sialylation, bisecting *N*-acetylglucosamine (GlcNAc), core fucosylation and mannose. The amounts of GP and IGP were normalized followed by log transformation and batch-effect was considered and corrected. The detailed structural and biological information of each GP and IGP was shown in **Supplementary Table S2**.

### Statistical analysis

Continuous variables adhering to the normal distribution were represented as mean and standard deviation (SD), and the differences between groups were tested by the independent ANOVA tests; otherwise, the median and interquartile range (P25, P75) were used, and the differences were explored by Kruskal-Wallis H tests. Categorical variables were presented as n (%), and the differences were tested by the chi-square tests. The box plots were used to show the differences of IgG GPs and IGPs among the controls, precancerosis and early ESCC.

The false discovery rate (FDR) correction was used to primarily identify the substantially increased or decreased IgG glycans and traits associated with ESCC. Then, the candidate glycans and traits selected above were finally confirmed using the stepwise ordinal logistics regression according to Akaike information criterion (AIC), which composed of an ESCC-related glycan score by the regression coefficients. The glycan score and its components were tested both in the discovery and validation population after the confounding covariates adjusted in three models: model 1 was unadjusted; model 2 was adjusted for age and sex; model 3 was further adjusted for BMI, hypertension, smoke, drink, education, income, marriage status and dietary habits. Formula of the ESCC-related glycan score was listed below:

Score = ∑(β_n_ × amounts of each IgG GP and IGP n), where β is the ordinal logistics coefficient.

The discriminative capacity of the proposed ESCC-related glycan score was illustrated using multi-class receiver operating characteristic (ROC) curve, and the average area under-the curve (AUC) value was provided. Significant differences in the proposed ESCC-related glycan score between different groups in the discovery and validation populations were subsequently assessed using DeLong’s test. The robustness of the ESCC-related glycan score was assessed using a bootstrap procedure (k=100). The bootstrap method was used to resample distinct data sets 100 times from the original data set, and the number of subjects in each resampled data set was set to be the same number as the sample size of the original data set. SNPs associated with the proposed ESCC-related glycan score were found out by Meta-analysis of the IgG N-glycosylation GWAS and were annotated. Gene Ontology (GO) and Kyoto Encyclopedia of Genes and Genomes (KEGG) Pathway enrichment analysis were carried as well as protein–protein interaction (PPI) network analysis to find potential hub genes. Finally, we validated the potential hub genes on The Cancer Genome Atlas (TCGA) and Genotype-Tissue Expression (GTEx) based on RNA sequence data. Detailed statistical methods are provided in the Supplementary material online. All statistical tests were two-sided at a significant level of 0.05, and the Benjamini-Hochberg method was applied to control the FDR for multiple hypothesis tests (34). All the analyses presented above were performed using the packages of ‘MASS’, ‘forestplot’, ‘multiROC’ in R software (version 4.0.0).

## Results

### Characteristics

In the discovery population, the median (P25, P75) age was 58.50 (54.00, 63.00), and 163 (46.84%) were males. In the validation population, the median (P25, P75) age was 60.00 (56.00, 64.00), and 65 (43.92%) were males. The characteristics were similar between the discovery and validation populations, except age as shown in **Supplementary Table S3**. There were no significant differences in sex, education level, marriage status, household income, BMI, hypertension, history, family history, dietary habits among the controls, precancerosis and early ESCC groups both in the discovery and validation populations, apart from age, smoking and drinking. The detailed distributions of the characteristics were shown in **Table 1**. In addition, the dietary habits, including the frequency of having pickled food, fried food, hot food, and mildew food, were similar among the controls, precancerosis and ESCC groups both discovery and validation populations (**Table 2**).

**Table 1:** Social-demographic characteristics in the discovery and validation populations.

	Discovery population (n=348)				Validation population (n=148)			
	Control (n=143)	Precancerosis (n=125)	Early ESCC (n=80)	P	Control (n=52)	Precancerosis (n=62)	Early ESCC (n=34)	P
**Age** (years)	57.00(54.00,61.00)	57.00(53.00,63.00)	64.00(57.75,66.25)	<0.001	59.50(56.75,62.00)	61.50(56.25,64.00)	59.50(55.25,65.75)	0.456
**Male**, n (%)	62(43.36)	62(49.60)	39(48.75)	0.550	25(48.08)	28(45.16)	12(35.29)	0.489
**Education level**, n (%)				0.117				0.574
Illiteracy	28(19.58)	26(20.80)	20(25.00)		16(30.77)	13(20.97)	5(14.71)	
Primary school	41(28.67)	38(30.40)	22(27.50)		19(36.54)	22(35.48)	11(32.35)	
Middle or high school	55(38.46)	46(36.80)	37(46.25)		14(26.92)	21(33.87)	14(41.18)	
Bachelor degree or above	19(13.29)	15(12.00)	1(1.25)		3(5.77)	6(9.68)	4(11.76)	
**Married**, n (%)	135(94.41)	113(90.40)	75(93.75)	0.419	49(94.23)	53(85.48)	32(94.12)	0.278
**Income** ≥ ¥50,000, n (%)	41(28.67)	35(28.00)	16(20.00)	0.328	20(38.46)	20(32.26)	7(20.59)	0.218
**BMI** ≥ 24.0 kg/m^2^, n (%)	74(51.75)	57(45.60)	35(43.75)	0.436	22(42.31)	24(38.71)	12(35.29)	0.805
**Hypertension**, n (%)	86(60.14)	86(68.80)	60(75.00)	0.064	32(61.54)	39(62.90)	28(82.35)	0.091
**History** *, n (%)	23(16.08)	17(13.60)	16(20.00)	0.477	7(13.46)	12(19.35)	6(17.65)	0.699
**Family history** *, n (%)	36(25.17)	34(27.20)	25(31.25)	0.620	11(21.15)	18(29.03)	9(26.47)	0.627
**Smoke status**, n (%)	60(41.96)	39(31.20)	25(31.25)	0.120	18(34.62)	19(30.65)	19(55.88)	0.043
**Drink status**, n (%)	44(30.77)	34(27.20)	24(30.00)	0.805	13(25.00)	12(19.35)	16(47.06)	0.013

Continuous variable is presented as the median (P_25_, P_75_) and examined by using Kruskal-Wallis H test; and categorical variables are presented as the number (percentage) and examined by using chi-square test.

* History refers to the gastroenteritis and peptic ulcer; Family history refers to esophageal cancer, gastric cancer and intestinal cancer.

**Table 2:** The dietary habits among the controls, precancerosis and early ESCC groups in the discovery and validation populations.

	Discovery population (n=348)			Validation population (n=148)		
	Control (n=143)	Precancerosis (n=125)	Early ESCC (n=80)	Control (n=52)	Precancerosis (n=62)	Early ESCC (n=34)
**Pickled**, n (%)	P = 0.878			P = 0.382		
Never	67(46.85)	59(47.20)	34(42.50)	25(48.08)	26(41.94)	15(44.12)
Seldom	27(18.88)	25(20.00)	14(17.50)	14(26.92)	10(16.13)	7(20.59)
Often	49(34.27)	41(32.80)	32(40.00)	13(25.00)	26(41.94)	12(35.29)
**Fried**, n (%)	P = 0.237			P = 0.493		
Never	62(43.36)	47(37.60)	43(53.75)	23(44.23)	23(37.10)	13(38.24)
Seldom	72(50.35)	67(53.60)	32(40.00)	23(44.23)	35(56.45)	20(58.82)
Often	9(6.29)	11(8.80)	5(6.25)	6(11.54)	4(6.45)	1(2.94)
**Hot** *, n (%)	P = 0.491			P = 0.689		
Never	80(55.94)	82(65.60)	51(63.75)	33(63.46)	33(53.23)	21(61.76)
Seldom	20(13.99)	11(8.80)	8(10.00)	7(13.46)	12(19.35)	7(20.59)
Often	43(30.07)	32(25.60)	21(26.25)	12(23.08)	17(27.42)	6(17.65)
**Mildew**, n (%)	P = 0.615			P = 0.246		
Never	141(98.60)	124(99.20)	78(97.50)	50(96.15)	61(98.39)	33(97.06)
Seldom	2(1.40)	1(0.80)	1(1.25)	2(3.85)	0(0.00)	0(0.00)
Often	0(0.00)	0(0.00)	1(1.25)	0(0.00)	1(1.61)	1(2.94)

Categorical variables are presented as the number (percentage) and analyzed using chi-square test.

* Hot refers to beverage or food with temperature above 65°C.

### Different IgG N-glycosylation patterns in ESCC, esophageal precancerosis, and controls

**Figure 2:**
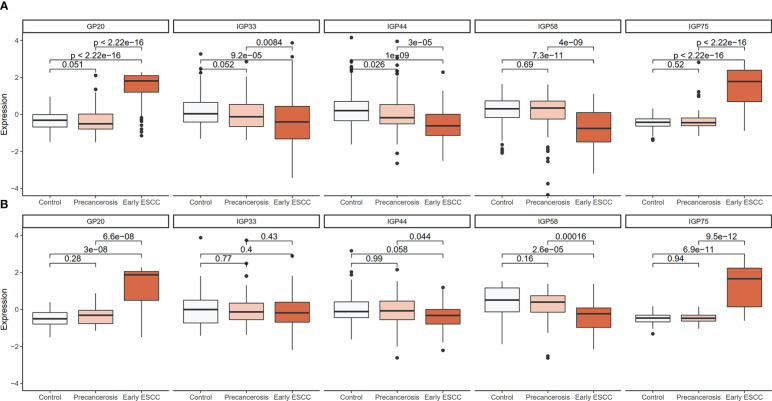
Distribution boxplot of differential GP and IGPs among the controls, precancerosis, and early ESCC groups. **(A)** The distribution boxplot of the IgG GP and IGPs in the discovery population; **(B)** The distribution boxplot of the IgG GP and IGPs in the validation population. The vertical position of each histogram represents the relative amount level of GP and IGPs.

The detailed distribution of IgG glycans and traits among the controls, precancerous and early ESCC groups were shown in **Supplementary Table S4**. A total of 7 GPs (GP3, GP6, GP12, GP13, GP17, GP20, GP23) and 11 IGPs (IGP30, IGP36, IGP37, IGP38, IGP46, IGP51, IGP52, IGP57, IGP73, IGP75, IGP77) substantially increased in the carcinogenesis progression of ESCC (**Supplementary Figure S2A**), while GP5 and 14 IGPs (IGP31, IGP33, IGP34, IGP43, IGP44, IGP47, IGP55, IGP56, IGP58, IGP60, IGP61, IGP62, IGP63, IGP76) showed negative association (**Supplementary Figure S2B**). After stepwise ordinal logistics regression, GP20 and 4 IGPs (IGP33, IGP44, IGP58, IGP75) retained in the final model and the AIC declined from 749.56 to 531.73. The distribution of these GP and IGPs were presented in **Figure 2**. In both the discovery and validation populations, compared with the control group, GP20 and IGP75 were elevated (P < 0.05); whereas IGP33, IGP44, and IGP58 were decreased in the early ESCC group. Similarly, GP20, IGP33, IGP44, IGP58 and IGP75 differed statistically between the early ESCC group and the precancerosis group. **Table 3** summarized the association of IgG glycans and traits with ESCC. In the discovery population, the adjusted ORs of GP20, IGP33, IGP44, IGP58, IGP75 were 4.03 (95% CI: 3.03-5.36, P<0.001), 0.69 (95% CI: 0.55-0.87, P<0.001), 0.56 (95% CI: 0.45-0.69, P<0.001), 0.52 (95% CI: 0.41-0.65, P<0.001), and 7.17 (95% CI: 4.77-10.79, P<0.001) respectively, while in the validation population, the adjusted OR were 7.41 (95% CI: 4.17-13.17, P<0.001), 0.66 (95% CI: 0.45-0.99, P<0.045), 0.60 (95% CI: 0.39-0.92, P<0.020), 0.48 (95% CI: 0.32-0.71, P<0.001), and 14.88 (95% CI: 5.75-38.47, P<0.001).

**Table 3:** Associations of the IgG GP and IGPs with carcinogenesis progression of ESCC by ordinal logistic models.

	Discovery population			Validation population		
	OR	95%CI	P	OR	95%CI	P
Model 1						
GP20	3.83	2.96-4.96	<0.001	5.20	3.27-8.27	<0.001
IGP33	0.66	0.53-0.81	<0.001	0.68	0.48-0.96	0.027
IGP44	0.57	0.46-0.70	<0.001	0.69	0.48-0.98	0.039
IGP58	0.53	0.42-0.65	<0.001	0.55	0.39-0.78	<0.001
IGP75	6.97	4.77-10.19	<0.001	9.83	4.66-20.75	<0.001
Model 2						
GP20	3.67	2.83-4.77	<0.001	5.20	3.26-8.29	<0.001
IGP33	0.67	0.54-0.83	<0.001	0.64	0.45-0.92	0.016
IGP44	0.57	0.46-0.70	<0.001	0.67	0.47-0.97	0.032
IGP58	0.53	0.43-0.66	<0.001	0.55	0.38-0.78	0.001
IGP75	6.85	4.64-10.11	<0.001	9.93	4.61-21.39	<0.001
Model 3						
GP20	4.03	3.03-5.36	<0.001	7.41	4.17-13.17	<0.001
IGP33	0.69	0.55-0.87	<0.001	0.66	0.45-0.99	0.045
IGP44	0.56	0.45-0.69	<0.001	0.60	0.39-0.92	0.020
IGP58	0.52	0.41-0.65	<0.001	0.48	0.32-0.71	<0.001
IGP75	7.17	4.77-10.79	<0.001	14.88	5.75-38.47	<0.001

Model 1: unadjusted; Model 2: adjusted for age, sex; Model 3: adjusted for age, sex, BMI, hypertension, smoke, drink, education level, income, marriage status, dietary habits.

### Construction and assessment of a glycan score for differentiating ESCC from esophageal precancerosis and controls

**Figure 3:**
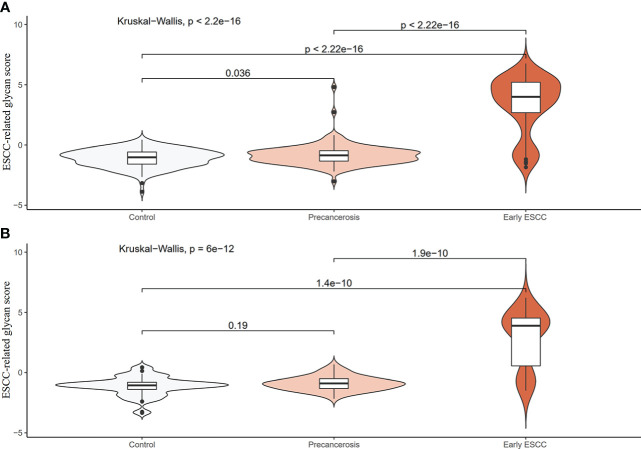
Distribution violin plot of the ESCC-related glycan score among the controls, precancerosis, and early ESCC groups. **(A)** The violin plot of the glycan score in the discovery population; **(B)** The violin plot of the glycan score in the validation population.

**Figure 4:**
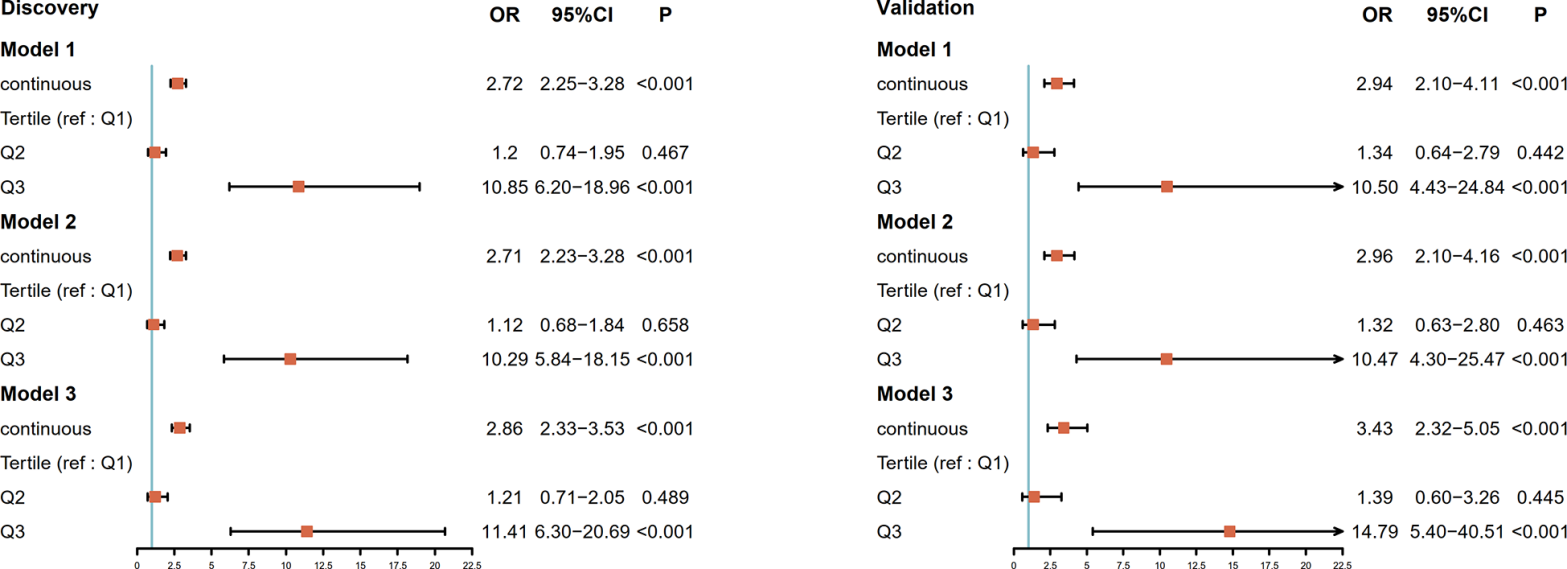
Forest plot for the association of the ESCC-related glycan score and progression of ESCC in the discovery population and the validation population. The ESCC-related glycan score estimates the magnitude of the effect as a continuous variable and tertile. The vertical line indicates no effect (odds ratio 1.0); horizontal lines indicate 95% confidence interval. Model 1: unadjusted; Model 2: adjusted for age, sex; Model 3: adjusted for age, sex, BMI, hypertension, smoke, drink, education level, income, marriage status, dietary habits; OR, odds ratio; CI, confidence interval; ref, reference.

**Figure 5:**
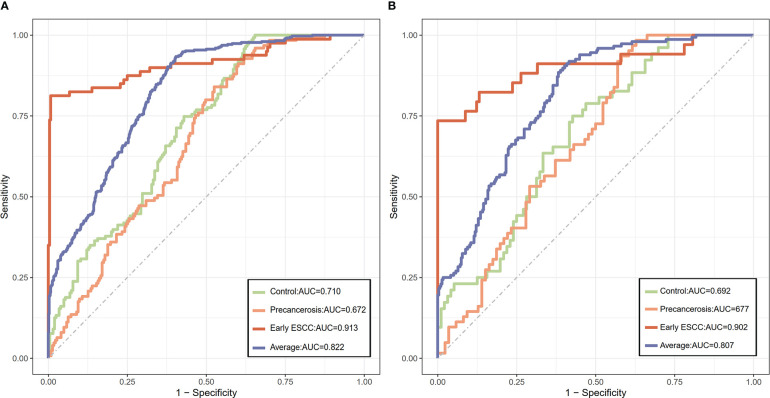
The discriminative capacity of the ESCC-related glycan score among the controls, precancerous, and early ESCC populations. **(A)** The ROC plot of the ESCC-related glycan score in the discovery population; **(B)** The ROC plot of the ESCC-related glycan score in the validation population.

We screened ESCC-related N-glycan alterations based on ordinal logistic regression analysis. Regression coefficients were used to estimate odds ratios for each of the independent variables. The mathematic formula named ESCC-related glycan score was constructed to differentiate ESCC from esophageal precancerosis and controls (ESCC-related glycan score = 0.612×GP20 - 0.357×IGP33 - 0.623×IGP44 - 0.439×IGP58 + 1.333×IGP75). The distinct distribution of the ESCC-related glycan score was shown in **Figure 3**. In both the discovery and validation populations, compared with precancerosis and controls, ESCC-related glycan score was elevated (P < 0.001) in the early ESCC group. In the discovery population, compared with the controls, ESCC-related glycan score was slightly increased (P < 0.05) while there was no difference in the validation population. After adjusting confounders including age, sex, BMI, hypertension, smoke, drink, education, income, marriage status and dietary habits (model 3), the ESCC-related glycan score showed significant association with the carcinogenesis progression of ESCC, and the adjusted ORs were 2.86 (95% CI: 2.33-3.53, P<0.001) in the discovery population, and 3.43 (95% CI: 2.32-5.05, P<0.001) in the validation population. Individuals in the highest tertile of the glycan score owned a higher risk compared with those in the lowest, and the adjusted ORs were 11.41 (95% CI: 6.30-20.69, P<0.001) and 14.79 (95% CI: 5.40-40.51, P<0.001), respectively, (**Figure 4**). **Figure 5** illustrated the multi-class ROC curves were of the ESCC-related glycan score for discriminating the controls, esophageal precancerosis and ESCC patients. Accordingly, the AUC value in the discrimination of the controls, esophageal precancerosis and early ESCC patients were 0.710 (95% CI: 0.656-0.775), 0.672 (95% CI: 0.625-0.735) and 0.913 (95% CI: 0.868-0.969) in the discovery population, and 0.692 (95% CI: 0.589-0.788), 0.677 (95% CI: 0.597-0.781) and 0.902 (95% CI: 0.824-0.982) in the validation population. The AUC value of early ESCC patients was significantly different from the controls (<0.01) and esophageal precancerosis (<0.001) in both discovery and validation populations. However, no statistically significant difference was found between the ROC curves of the controls and esophageal precancerosis in the validation populations (p>0.05) (**Supplementary Table S5**). The ESCC-related glycan score achieved an average AUC of 0.822 (95% CI: 0.786-0.849) and 0.807 (95% CI: 0.758-0.864), respectively. The results after combining the two populations were similar to each single population.

After linkage disequilibrium, we found 27 SNPs were associated with the proposed ESCC-related glycan score and 15 of them could be annotated to functional genes (**Supplementary Table S6**). In total, the genes were significantly enriched in 12 different GO gene sets and 2 different KEGG gene sets (**Supplementary Figure S3**), and construct a PPI network topology includes 595 nodes and 723 edges (**Supplementary Figure S4**). Based on the node degree score, the top 6 genes, including SMARCB1, IKZF1, RUNX1, TAB1, RUNX3 and B4GALT1 were considered as potential hub genes. After validation on RNA sequence data in the database online, these 6 genes were differently expressed in ESCC and normal tissues (**Supplementary Figure S5**), which may be the corroborative evidence of our study.

## Discussion

In this current study, we investigated the association of IgG N-glycosylation profiles and the carcinogenesis progression of ESCC. IgG N-glycans (GP20) and the derived traits (IGP33, IGP44, IGP58, IGP75) were primarily selected and validated to be associated with different stages of ESCC. Specific IgG N-glycosylation pattern participates in the carcinogenesis progression of ESCC, and the proposed ESCC-related glycan score could be a novel indicator. Variation in the fucosylated glycans and the suppressed mannose level, reflected by the altered glycans and traits, could be potential intervention target for ESCC. In addition, an ESCC-related glycan score was composed in this study, which achieved a high AUC value to discriminate different stages of ESCC. Besides, SMARCB1, IKZF1, RUNX1, TAB1, RUNX3, B4GALT1 were considered as potential hub genes of the proposed ESCC-related glycan score.

In our study, we found that GP20 and IGP75 was positively associated with ESCC progression, while IGP33, IGP44 and IGP58 was negatively associated (GP20: digalactosylated monosialylated biantennary with core and antennary fucose; IGP33: the ratio of all fucosylated monosyalilated and disialylated structures; IGP44: the proportion of high mannose glycan structures in total neutral IgG glycans; IGP58: the percentage of all fucosylated structures in total neutral IgG glycans; IGP75: the incidence of bisecting GlcNAc in all fucosylated digalactosylated structures in total neutral IgG glycans). These results above revealed a glycosylation pattern of increased digalactosylated biantennary glycan, the incidence of bisecting GlcNAc in all fucosylated digalactosylated glycans, and decreased high mannose glycan, fucosylated glycan, the ratio of all fucosylated monosyalilated and disialylated glycan among ESCC.

These finding were largely in consistent with previous studies. Liu et al. reported a significantly decreased of mannose glycan in patients with colorectal cancer (35) and we found a decrease of glycans with mannosylation in precancerous lesions and early esophageal cancer. It was also observed that, mannose glycan was distinctively decreased in breast cancer relative to control in total mouse serum proteins, demonstrating that mannosylation may play an important role in cancer progression not only in human but also in other animals (36). Removal of mannose sugar residues resulting in conformational changes in Cgamma2 domain affected the structure and function of IgG-Fc fragments (37), showing the importance of mannosylation. Gornik et al. found that IgG would activate complement and ADCC, and promote anti-inflammatory activity according to the extent of galactosylation and fucosylation of its glycans (38). Sialylation plays a crucial role in the inflammatory potential of IgG. Addition of sialic acid to IgG would decrease its binding to Fcγ receptors, and converts the function from pro- to anti-inflammatory (39). Sethi et al. reported that the expression levels of disialylation was higher in mid-and late-stage colorectal tumors than in early tumors (29) and we found the ratio of all fucosylated monosyalilated and disialylated structures was negatively associated with ESCC progression. Similar to the critical role of sialylated glycan in the regulation of inflammatory action, the fucosylated glycan can also enhance or inhibit IgG-mediated ADCC (40). Liu et al. reported that fucosylation and sialylation were associated with lung tumor cell growth and malignancy (41). Some previous studies pointed out that the decrease of fucosylated glycan was probably associated with colorectal cancer progression (35, 42), and we found a decrease of glycans with fucosylation in ESCC progression. Therefore, it is of significance to reveal the changes of IgG N-glycans abundance, and to explore the profiling of IgG N-glycans as potential biomarker for early detection of ESCC.

Our study found SMARCB1, IKZF1, RUNX1, TAB1, RUNX3 and B4GALT1 as potential hub genes for the proposed ESCC-related glycan score, which were in agreement with previous studies. B4GALT1, IKZF1, TAB1 and SMARCB1 are reported to associate with IgG N-glycosylation show pleiotropy with autoimmune diseases and haematological cancers (43), while Shen et al. used multivariate methods in a genome-wide association study certified B4GALT1 and SMARCB1 are related to IgG N-glycosylation (44). TAB1 has also been reported associated with the progression and prognosis of esophageal cancer (45). RUNX3 encodes for a transcription factor of the runt domain-containing family. Methylation of RUNX3 promoters has an impact on cancers (46–49) and B-cell maturation (50). By influencing T-cell differentiation, RUNX3 is likely to indirectly affect the glycosylation of antibodies produced by B-cells. IKZF1, attributed to the enzymes of the Ikaros family, can also alter the differentiation process of T-cells (51, 52). Klarić et al. confirmed *in vitro* that knockdown of IKZF1 decreases the expression of fucosyltransferase FUT8, resulting in increased levels of fucosylated glycans, and suggest that RUNX1 and RUNX3, together with SMARCB1, regulate expression of glycosyltransferase MGAT3 (53).

In this study we explored the significant differences in IgG N-glycosylation profile among early ESCC, esophageal precancerosis, and the controls. To our knowledge, this is the first attempt aiming at the association of IgG N-glycans biomarkers with the carcinogenesis progression of ESCC. The identified glycans and proposed glycan score were validated in another population. However, the limitations should be addressed. First, the sample size was relatively small causing an inadequate statistical power. Second, this was a population-based cross-sectional study, hence, no causal relationships or pathophysiological inferences were available, basic experiments *in vivo* or *in vitro* will be conducted to confirm the association of IgG N-glycans biomarkers with the carcinogenesis progression of ESCC. Third, our study was based on two Chinese populations, more collaborations are needed to validate the generalizability of the observed results for other ethnic groups. Fourth, the identification and quantification of glycans were by HPLC in our study, although glycan standards were used, additional cross validation with other techniques, e.g. mass spectrometry, lectin array will be performed in our further research.

## Conclusions and perspectives

In summary, we have performed the first analysis so far to identify the association of IgG N-glycans biomarkers with the carcinogenesis progression of ESCC. In this study, GP20, IGP33, IGP44, IGP58, IGP75 are significantly associated with the carcinogenesis progression of ESCC, and the proposed glycan score is a novel indicator for different progressive stages. In addition, the variation of fucosylation level and the suppressed mannose level could provide potential therapeutic intervention targets. These findings support the potential utility of glycomics in the ESCC related personalized therapy. The mechanism studies about the biological or pathological function of the fucosylated protein and mannosed protein in the carcinogenesis of ESCC and other cancers are of paramount importance. The experiment on mice after knocking out the corresponding genes of glycosyltransferase and glycosylhydrolase regulating the fucosylation and mannose levels are the next step for our study to validate the effect of IgG N-glycan patterns in the carcinogenesis of ESCC. Future studies on larger cohorts from diverse populations are expected for the validation of these observed associations.

## Data availability statement

The raw data supporting the conclusions of this article will be made available by the authors, without undue reservation.

## Ethics statement

The studies involving human participants were reviewed and approved by the Independent Ethics Committee of National Cancer Center/Cancer Hospital, Chinese Academy of Medical Sciences and Peking Union Medical College and the Ethics Committees of Capital Medical University. The patients/participants provided their written informed consent to participate in this study.

## Author contributions

XG and GW contributed to conception and design of the study. DZ, ZYZ, YQL, ZZ, ZW, JZ, YuL, YoL, and HZ collected the data. HP, ZL, WF, XTL, and YXL performed the statistical analysis. HP and ZW wrote the first draft of the manuscript. LT, XW, XY, and FZ wrote sections of the manuscript. All authors contributed to the article and approved the submitted version.

## Supplementary material

Supplementary Figure 1 UPLC analysis of the IgG glycome. IgG glycome was separated into 24 chromatographic peaks by hydrophilic interaction chromatography.

Supplementary Figure 2 Distribution boxplot of substantially changed GPs and IGPs among the controls, precancerosis, and early ESCC groups. A: The distribution boxplot of the substantially increased IgG GPs and IGPs among the controls, precancerosis, and early ESCC groups; B: The distribution boxplot of the substantially decreased IgG GPs and IGPs among the controls, precancerosis, and early ESCC groups. The vertical position of each histogram represents the relative amount level of GPs and IGPs.

Supplementary Figure 3 GO and KEGG gene-set enrichment analysis. Adjusted p-value*: P*-value was adjusted by Benjamini-Hochberg method.

Supplementary Figure 4 PPI network. The edges between 2 nodes represent the gene-gene interactions. The size of the nodes corresponding to each gene were determined according to the degree of interaction.

Supplementary Figure 5 Distribution boxplot of gene expression between the normal and ESCC groups. The vertical position of each histogram represents the relative expression level of genes.
