# Supplementary material for: Identification and validation of IgG N-glycosylation biomarkers of esophageal carcinoma
Source: Front Immunol. 2023 Mar 14;14:981861. doi: 10.3389/fimmu.2023.981861 (PMC10043232; doi:10.3389/fimmu.2023.981861)
Supplement: Supplementary file 1 [file DataSheet_1.docx]

**Supplementary materials**

**Supplemental Methods**

**Statistical Analysis**

We selected genetic variants that were reported to be significantly associated with IgG N-glycosylation traits that are associated with the proposed ESCC-related glycan score in our study at genomewide level (P-value of association ≤ 5 × 10−8) in previous Meta-analysis of the IgG N-glycosylation GWAS [1] and performed clumping of the selected SNPs within a window of 500 Kb and the lowest P value having linkage disequilibrium r2 <0.001 to obtain a set of independent SNPs. Then, SNPs were annotated with VEP [2] to obtain the functional effect of associated variants (Supplementary Table S6). To obtain insights into the biological pathways that these genes are involved in, we performed FUMA’s GENE2FUNC [3] for Gene Ontology (GO) and Kyoto Encyclopedia of Genes and Genomes (KEGG). An online tool NetworkAnalyst 3.0 (https://www.networkanalyst.ca/) was used to analyze protein interactions based on STRING [4] and the protein-protein interaction (PPI) network was visualized by Cytoscape. A confidence score >0.40 was used to screen the protein–protein interaction pairs. The top 6 genes were taken to as potential hub genes based on the node degree score. Finally, we validated the potential hub genes on The Cancer Genome Atlas (TCGA) and Genotype-Tissue Expression (GTEx), including 666 normal tissue and 182 ESCC tissue. RNA sequence data in TPM format of TCGA and GTEx uniformly processed by Toil process [5].

**REFERENCES**

[1] Klarić L, Tsepilov YA, Stanton CM, Mangino M, Sikka TT, Esko T, Pakhomov E, Salo P, Deelen J, McGurnaghan SJ, Keser T, Vučković F, Ugrina I, Krištić J, Gudelj I, Štambuk J, Plomp R, Pučić-Baković M, Pavić T, Vilaj M, Trbojević-Akmačić I, Drake C, Dobrinić P, Mlinarec J, Jelušić B, Richmond A, Timofeeva M, Grishchenko AK, Dmitrieva J, Bermingham ML, Sharapov SZ, Farrington SM, Theodoratou E, Uh HW, Beekman M, Slagboom EP, Louis E, Georges M, Wuhrer M, Colhoun HM, Dunlop MG, Perola M, Fischer K, Polasek O, Campbell H, Rudan I, Wilson JF, Zoldoš V, Vitart V, Spector T, Aulchenko YS, Lauc G, Hayward C. Glycosylation of immunoglobulin G is regulated by a large network of genes pleiotropic with inflammatory diseases. Sci Adv. 2020, 6(8):eaax0301.

[2] McLaren W, Gil L, Hunt SE, Riat HS, Ritchie GR, Thormann A, Flicek P, Cunningham F. The Ensembl Variant Effect Predictor. Genome Biol. 2016, 17(1):122.

[3] Watanabe K, Taskesen E, van Bochoven A, Posthuma D. Functional mapping and annotation of genetic associations with FUMA. Nat Commun. 2017, 8(1):1826.

[4] Szklarczyk D, Franceschini A, Wyder S, Forslund K, Heller D, Huerta-Cepas J, Simonovic M, Roth A, Santos A, Tsafou KP, Kuhn M, Bork P, Jensen LJ, von Mering C. STRING v10: protein-protein interaction networks, integrated over the tree of life. Nucleic Acids Res. 2015, 43(Database issue): D447-52.

[5] Vivian J, Rao AA, Nothaft FA, Ketchum C, Armstrong J, Novak A, Pfeil J, Narkizian J, Deran AD, Musselman-Brown A, Schmidt H, Amstutz P, Craft B, Goldman M, Rosenbloom K, Cline M, O'Connor B, Hanna M, Birger C, Kent WJ, Patterson DA, Joseph AD, Zhu J, Zaranek S, Getz G, Haussler D, Paten B. Toil enables reproducible, open source, big biomedical data analyses. Nat Biotechnol. 2017, 35(4):314-316. 

**Supplementary Table S1**

The detailed information of questionnaire in this study.

**Survey on related factors of esophageal cancer in high risk region**

Identification Card Number:

Dear all:

The mortality and mortality of esophageal cancer were relatively high, with 5.90 new onsets and 5.48 deaths per 100000 persons worldwide, ranking 7th and 6th respectively among all cancers. In China, there are some high incidence regions of esophageal cancer, involving Feicheng City and Wuwei City. In order to investigate the related factors, Capital Medical University together with two designated local hospitals carried out this survey and please fill in the following questionnaire seriously. This survey is based on the National Key R&D Program of China and all the contents of questionnaire will be kept confidential. Thank you for your cooperation.

**Part A: Demographic characteristics**

1. name: 2. sex: ①male ②female 3. birth:

4. race: 5. height: cm 6. weight: kg

7. marriage: ①married ②others

8. income: ①≥50,000 per capita per year ②<50,000 per capita per year

9. education: ①illiteracy ②primary ③middle or high school ④bachelor or above

10. residence:

**Part B: Life behavior and disease history**

1. smoke: ①yes (at least one cigarette on daily average in past year) ②no (never or abandon)

2. drink: ①yes (at least 100 ml on daily consumption of 50% alcohol) ②no (never or abandon)

3. History of gastrointestinal disease diseases involving gastroenteritis and peptic ulcer:

①yes ②no

4. Family history of gastrointestinal cancer involving esophageal cancer, gastric cancer, intestinal cancer: ①yes ②no

5. Do you have the following diseases, involving mental illness, infectious disease, autoimmune diseases or and other malignant cancers: ①yes ②no

6. Are you in pregnancy or lactation: ①yes ②no

**Part C: Dietary habit**

Dietary frequency of vegetables/friuts: ①never ②seldom ③often

Dietary frequency of pickled food: ①never ②seldom ③often

Dietary frequency of fried food: ①never ②seldom ③often

Dietary frequency of hot food: ①never ②seldom ③often

Dietary frequency of mildew food: ①never ②seldom ③often

**Supplementary Table S2**

The structures and descriptions of the IgG glycans and derived traits.

Structure abbreviations: F, core fucose; A, number of antenna's; B, bisecting GlcNAc; M, number of mannose residues; Gx, number of galactoses; Sx, number of sialic acids linked to galactose; n, neutral glycans.

|  | Structure | Description |
| --- | --- | --- |
| **Glycan peak** |  |  |
| GP1 | FA1 | Monoantennary glycan with core fucose |
| GP2 | A2 | Biantennary glycan |
| GP3 | - | Structure not determined |
| GP4 | FA2 | Monoantennary glycan with core fucose |
| GP5 | M5 | High mannose glycan |
| GP6 | FA2B | Biantennary glycan with bisecting GlcNAc and core fucose |
| GP7 | A2G1 | Monogalactosylated biantennary glycan |
| GP8 | FA2[6]G1 | Monogalactosylated biantennary glycan with 1-6 linkages with core fucose |
| GP9 | FA2[3]G1 | Monogalactosylated biantennary glycan with 1-3 linkages with core fucose |
| GP10 | FA2[6]BG1 | Monogalactosylated biantennary glycan with 1-6 linkages with bisecting GlcNAc and core fucose |
| GP11 | FA2[3]BG1 | Monogalactosylated biantennary glycan with 1-3 linkages with bisecting GlcNAc and core fucose |
| GP12 | A2G2 | Digalactosylated biantennary glycan |
| GP13 | A2BG2 | Digalactosylated biantennary glycan with bisecting GlcNAc |
| GP14 | FA2G2 | Digalactosylated biantennary glycan with core fucose |
| GP15 | FA2BG2 | Digalactosylated biantennary glycan with bisecting GlcNAc and core fucose |
| GP16 | FA2G1S1 | Monogalactosylated monosialylated biantennary glycan with core fucose |
| GP17 | A2G2S1 | Digalactosylated monosialylated biantennary glycan |
| GP18 | FA2G2S1 | Digalactosylated monosialylated biantennary glycan with core fucose |
| GP19 | FA2BG2S1 | Digalactosylated monosialylated biantennary glycan with bisecting GlcNAc and core fucose |
| GP20 | FA2FG2S1 | Digalactosylated monosialylated biantennary with core and antennary fucose |
| GP21 | A2G2S2 | Digalactosylated disialylated biantennary glycan |
| GP22 | A2BG2S2 | Digalactosylated disialylated biantennary glycan with bisecting GlcNAc |
| GP23 | FA2G2S2 | Digalactosylated disialylated biantennary glycan with core fucose |
| GP24 | FA2BG2S2 | Digalactosylated disialylated biantennary glycan with bisecting GlcNAc and core fucose |
| **Derived trait** |  |  |
| IGP24 | FGS/(FG+FGS) | Sialylation of fucosylated galactosylated structures without bisecting GlcNAc |
| IGP25 | FBGS/(FBG+FBGS) | Sialylation of fucosylated galactosylated structures with bisecting GlcNAc |
| IGP26 | FGS/(F+FG+FGS) | Sialylation of all fucosylated structures without bisecting GlcNAc |
| IGP27 | FBGS/(FB+FBG+FBGS) | Sialylation of all fucosylated structures with bisecting GlcNAc |
| IGP28 | FG1S1/(FG1+FG1S1) | Monosialylation of fucosylated monogalactosylated structures |
| IGP29 | FG2S1/(FG2+FG2S1+FG2S2) | Monosialylation of fucosylated digalactosylated structures |
| IGP30 | FG2S2/(FG2+FG2S1+FG2S2) | Disialylation of fucosylated digalactosylated structures |
| IGP31 | FBG2S1/(FBG2+FBG2S1+FBG2S2) | Monosialylation of fucosylated digalactosylated structures with bisecting GlcNAc |
| IGP32 | FBG2S2/(FBG2+FBG2S1+FBG2S2) | Disialylation of fucosylated digalactosylated structures with bisecting GlcNAc |
| IGP33 | FtotalS1/FtotalS2 | Ratio of all fucosylated (+/- bisecting glynac) monosyalilated and disialylated structures |
| IGP34 | FS1/FS2 | Ratio of fucosylated (without bisecting GlcNAc) monosialylated and disialylated structures |
| IGP35 | FBS1/FBS2 | Ratio of fucosylated (with bisecting GlcNAc) monosialylated and disialylated structures |
| IGP36 | FBStotal/FStotal | Ratio of all fucosylated sialylated structures with and without bisecting GlcNAc |
| IGP37 | FBS1/FS1 | Ratio of fucosylated monosialylated structures with and without bisecting GlcNAc |
| IGP38 | FBS1/(FS1+FBS1) | The incidence of bisecting GlcNAc in all fucosylated monosialylated structures |
| IGP39 | FBS2/FS2 | Ratio of fucosylated disialylated structures with and without bisecting GlcNAc |
| IGP40 | FBS2/(FS2+FBS2) | The incidence of bisecting GlcNAc in all fucosylated disialylated structures |
| IGP41 | GP1n | Monoantennary glycan with core fucose structures in total neutral IgG glycans |
| IGP42 | GP2n | Biantennary glycan structures in total neutral IgG glycans |
| IGP43 | GP4n | Biantennary glycan with core fucose structures in total neutral IgG glycans |
| IGP44 | GP5n | High mannose glycan structures in total neutral IgG glycans |
| IGP45 | GP6n | Biantennary glycan with bisecting GlcNAc and core fucose structures in total neutral IgG glycans |
| IGP46 | GP7n | Monogalactosylated biantennary glycan structures in total neutral IgG glycans |
| IGP47 | GP8n | Monogalactosylated biantennary glycan with 1-6 linkages structures in total neutral IgG glycans |
| IGP48 | GP9n | Monogalactosylated biantennary glycan with 1-3 linkages structures in total neutral IgG glycans |
| IGP49 | GP10n | Monogalactosylated biantennary glycan with 1-6 linkages with bisecting GlcNAc structures in total neutral IgG glycans |
| IGP50 | GP11n | Monogalactosylated biantennary glycan with 1-3 linkages with bisecting GlcNAc structures in total neutral IgG glycans |
| IGP51 | GP12n | Digalactosylated biantennary glycan structures in total neutral IgG glycans |
| IGP52 | GP13n | Digalactosylated biantennary glycan with bisecting GlcNAc structures in total neutral IgG glycans |
| IGP53 | GP14n | Digalactosylated biantennary glycan with core fucose structures in total neutral IgG glycans |
| IGP54 | GP15n | Digalactosylated biantennary glycan with bisecting GlcNAc and core fucose in total neutral IgG glycans |
| IGP55 | G0n | Monogalactosylated and digalactosylated structures in total neutral IgG glycans |
| IGP56 | G1n | Monogalactosylated structures in total neutral IgG glycans |
| IGP57 | G2n | Digalactosylated structures in total neutral IgG glycans |
| IGP58 | Fn total | All fucosylated (+/- bisecting GlcNAc) structures in total neutral IgG glycans |
| IGP59 | FG0n total/G0n | Fucosylation of agalactosylated structures |
| IGP60 | FG1n total/G1n | Fucosylation of monogalactosylated structures |
| IGP61 | FG2n total /G2n | Fucosylation of digalactosylated structures |
| IGP62 | Fn | Fucosylated (without bisecting GlcNAc) structures in total neutral IgG glycans |
| IGP63 | FG0n/G0n | Fucosylation (without bisecting GlcNAc) of agalactosylated structures |
| IGP64 | FG1n/G1n | Fucosylation (without bisecting GlcNAc) of monogalactosylated structures |
| IGP65 | FG2n/G2n | Fucosylation (without bisecting GlcNAc) of digalactosylated structures |
| IGP66 | FBn | Fucosylated (with bisecting GlcNAc) structures in total neutral IgG glycans |
| IGP67 | FBG0n/G0n | Fucosylation (with bisecting GlcNAc) of agalactosylated structures |
| IGP68 | FBG1n/G1n | Fucosylation (with bisecting GlcNAc) of monogalactosylated structures |
| IGP69 | FBG2n/G2n | Fucosylation (with bisecting GlcNAc) of digalactosylated structures |
| IGP70 | FBn/Fn | Ratio of fucosylated structures with and without bisecting GlcNAc |
| IGP71 | FBn/Fn total | The incidence of bisecting GlcNAc in all fucosylated structures in total neutral IgG glycans |
| IGP72 | Fn/(Bn + FBn) | Ratio of fucosylated non-bisecting GlcNAc structures and all structures with bisecting GlcNAc |
| IGP73 | Bn/(Fn + FBn) | Ratio of structures with bisecting GlcNAc and all fucosylated structures (+/- bisecting GlcNAc) |
| IGP74 | FBG2n/FG2n | Ratio of fucosylated digalactosylated structures with and without bisecting GlcNAc |
| IGP75 | FBG2n /(FG2n + FBG2n) | The incidence of bisecting GlcNAc in all fucosylated digalactosylated structures in total neutral IgG glycans |
| IGP76 | FG2n/(BG2n + FBG2n) | Ratio of fucosylated digalactosylated non-bisecting GlcNAc structures and all digalactosylated structures with bisecting GlcNAc |
| IGP77 | BG2n/(FG2n + FBG2n) | Ratio of digalactosylated structures with bisecting GlcNAc and all fucosylated digalactosylated structures (+/- bisecting GlcNAc) |

**Supplementary Table S3**

The characteristics in the discovery and validation populations

| Variables | All | Discovery | Validation | P |
| --- | --- | --- | --- | --- |
|  | (n=496) | (n=348) | (n=148) |  |
| **Age** (years) | 59.00(55.00, 63.00) | 58.50(54.00, 63.00) | 60.00(56.00, 64.00) | 0.039 |
| **Sex**, n (%) |  |  |  | 0.618 |
| Male | 228(45.97) | 163(46.84) | 65(43.92) |  |
| Female | 268(54.03) | 185(53.16) | 83(56.08) |  |
| **Education level**, n (%) |  |  |  | 0.432 |
| Illiteracy | 108(21.77) | 74(21.26) | 34(22.97) |  |
| Primary school | 153(30.85) | 101(29.02) | 52(35.14) |  |
| Middle or high school | 187(37.70) | 138(39.66) | 49(33.11) |  |
| Bachelor degree or above | 48(9.68) | 35(10.06) | 13(8.78) |  |
| **Marital status**, n (%) |  |  |  | 0.497 |
| Others | 39(7.86) | 25(7.18) | 14(9.46) |  |
| Married | 457(92.14) | 323(92.82) | 134(90.54) |  |
| **Household income**, n (%) |  |  |  | 0.272 |
| <¥50,000 | 357(71.98) | 256(73.56) | 101(68.24) |  |
| ≥¥50,000 | 139(28.02) | 92(26.44) | 47(31.76) |  |
| **BMI**, n (%) |  |  |  | 0.100 |
| BMI<24 kg/m^2^ | 272(54.84) | 182(52.30) | 90(60.81) |  |
| BMI≥24 kg/m^2^ | 224(45.16) | 166(47.70) | 58(39.19) |  |
| **Hypertension**, n (%) |  |  |  | 0.999 |
| No | 165(33.27) | 116(33.33) | 49(33.11) |  |
| Yes | 331(66.73) | 232(66.67) | 99(66.89) |  |
| **History** ‡, n (%) |  |  |  | 0.930 |
| No | 415(83.67) | 292(83.91) | 123(83.11) |  |
| Yes | 81(16.33) | 56(16.09) | 25(16.89) |  |
| **Family history** ‡, n (%) |  |  |  | 0.793 |
| No | 363(73.19) | 253(72.70) | 110(74.32) |  |
| Yes | 133(26.81) | 95(27.30) | 38(25.68) |  |
| **Smoke**, n (%) |  |  |  | 0.715 |
| No | 316(63.71) | 224(64.37) | 92(62.16) |  |
| Yes | 180(36.29) | 124(35.63) | 56(37.84) |  |
| **Drink**, n (%) |  |  |  | 0.800 |
| No | 353(71.17) | 246(70.69) | 107(72.30) |  |
| Yes | 143(28.83) | 102(29.31) | 41(27.70) |  |
| **Diatery habits** |  |  |  |  |
| **Pickled food**, n (%) |  |  |  | 0.877 |
| Never | 226(45.56) | 160(45.98) | 66(44.59) |  |
| Seldom | 97(19.56) | 66(18.97) | 31(20.95) |  |
| Often | 173(34.88) | 122(35.06) | 51(34.46) |  |
| **Fried food**, n (%) |  |  |  | 0.731 |
| Never | 211(42.54) | 152(43.68) | 59(39.86) |  |
| Seldom | 249(50.20) | 171(49.14) | 78(52.70) |  |
| Often | 36(7.26) | 25(7.18) | 11(7.43) |  |
| **Hot food***, n (%) |  |  |  | 0.141 |
| Never | 300(60.48) | 213(61.21) | 87(58.78) |  |
| Seldom | 65(13.10) | 39(11.21) | 26(17.57) |  |
| Often | 131(26.41) | 96(27.59) | 35(23.65) |  |
| **Mildew food**, n (%) |  |  |  | 0.359 |
| Never | 487(98.19) | 343(98.56) | 144(97.30) |  |
| Seldom | 6(1.21) | 4(1.15) | 2(1.35) |  |
| Often | 3(0.60) | 1(0.29) | 2(1.35) |  |
| **Pathological type**, n (%) |  |  |  | 0.303 |
| Normal | 56 (11.3) | 41 (11.8) | 15 (10.1) |  |
| Oesophagitis | 139 (28.0) | 102 (29.3) | 37 (25.0) |  |
| Mild atypical hyperplasia | 164 (33.1) | 114 (32.8) | 50 (33.8) |  |
| Moderate atypical hyperplasia | 23 (4.6) | 11 (3.2) | 12 (8.1) |  |
| Severe atypical hyperplasia | 28 (5.6) | 19 (5.5) | 9 (6.1) |  |
| Mucosal carcinoma | 76 (15.3) | 55 (15.8) | 21 (14.2) |  |
| Submucosal carcinoma | 10 (2.0) | 6 (1.7) | 4 (2.7) |  |
| **Outcome**, n (%) |  |  |  | 0.383 |
| Control | 195 (39.3) | 143 (41.1) | 52 (35.1) |  |
| Precancerosis | 187 (37.7) | 125 (35.9) | 62 (41.9) |  |
| Early ESCC | 114 (23.0) | 80 (23.0) | 34 (23.0) |  |

Continuous variable is presented as the median (P_25_, P_75_) and examined by using Mann-Whitney U test; and categorical variables are presented as the number (percentage) and examined by using χ^2^ test.

Abbreviations: BMI, Body mass index

‡ History refers to the gastroenteritis and peptic ulcer; Family history refers to esophageal cancer, gastric cancer and intestinal cancer.

* Hot food refers to beverage or food with temperature above 65 ℃.

**Supplementary Table S4**

Distribution of IgG glycans and traits among the controls, precancerous and early ESCC populations

|  | **Control**  **(n=195)** | **Precancerosis**  **(n=187)** | **Early ESCC**  **(n=114)** | ***P*** | **Adj. *P*** |
| --- | --- | --- | --- | --- | --- |
| GP1 | -0.11(-0.83, 0.57) | -0.23(-0.83, 0.47) | 0.47(-0.32, 1.35) | 5.23E-07 | 8.68E-07 |
| GP2 | 0.02(-0.78, 0.61) | -0.04(-0.76, 0.63) | 0.47(-0.22, 1.07) | 9.61E-05 | 1.47E-04 |
| GP3 | -0.27(-0.80, 0.32) | -0.22(-0.93, 0.46) | 0.73(-0.26, 1.30) | 2.71E-11 | 5.15E-11 |
| GP4 | 0.11(-0.58, 0.76) | -0.04(-0.68, 0.66) | 0.06(-0.61, 0.66) | 7.37E-01 | 7.37E-01 |
| GP5 | 0.15(-0.41, 0.68) | -0.11(-0.59, 0.46) | -0.41(-1.02, 0.12) | 3.10E-07 | 5.26E-07 |
| GP6 | -0.01(-0.69, 0.62) | 0.10(-0.60, 0.60) | 0.23(-0.34, 0.85) | 2.30E-02 | 2.80E-02 |
| GP7 | -0.18(-0.84, 0.38) | -0.18(-0.64, 0.36) | 0.86(-0.25, 1.58) | 8.76E-13 | 1.85E-12 |
| GP8 | 0.28(0.20, 0.35) | 0.25(0.18, 0.34) | -0.11(-1.16, 0.28) | 2.91E-14 | 6.69E-14 |
| GP9 | -0.17(-0.53, 0.18) | -0.24(-0.73, 0.17) | 0.95(-0.02, 1.95) | 1.10E-18 | 3.30E-18 |
| GP10 | -0.19(-0.77, 0.25) | -0.24(-0.76, 0.26) | 0.88(-0.16, 2.06) | 1.59E-15 | 4.00E-15 |
| GP11 | -0.34(-0.61, -0.13) | -0.39(-0.64, -0.18) | 1.77(-0.08, 2.41) | 1.00E-20 | 3.39E-20 |
| GP12 | -0.09(-0.87, 0.45) | -0.05(-0.59, 0.55) | 0.67(-0.60, 1.47) | 3.95E-06 | 6.42E-06 |
| GP13 | -0.40(-0.70, 0.14) | -0.34(-0.70, -0.02) | 1.15(-0.14, 1.93) | 8.00E-20 | 2.60E-19 |
| GP14 | 0.29(0.07, 0.50) | 0.30(0.06, 0.52) | -0.19(-1.27, 0.20) | 1.01E-18 | 3.15E-18 |
| GP15 | -0.40(-0.62, -0.20) | -0.42(-0.62, -0.20) | 1.63(0.39, 2.44) | 1.00E-20 | 3.39E-20 |
| GP16 | 0.17(-0.30, 0.67) | 0.26(-0.32, 0.59) | -0.21(-1.13, 0.59) | 3.06E-03 | 4.19E-03 |
| GP17 | -0.20(-0.62, 0.19) | -0.20(-0.51, 0.40) | 0.67(0.36, 1.05) | 3.96E-15 | 9.35E-15 |
| GP18 | 0.24(-0.10, 0.62) | 0.29(-0.10, 0.60) | -0.38(-1.82, 0.30) | 2.18E-11 | 4.25E-11 |
| GP19 | 0.12(-0.20, 0.55) | 0.18(-0.24, 0.65) | -0.09(-1.19, 0.52) | 6.77E-03 | 8.52E-03 |
| GP20 | -0.37(-0.68, -0.05) | -0.43(-0.80, -0.01) | 1.84(1.09, 2.09) | 1.00E-20 | 3.39E-20 |
| GP21 | -0.28(-0.71, 0.21) | -0.22(-0.60, 0.24) | 1.03(-0.08, 1.78) | 1.54E-15 | 4.00E-15 |
| GP22 | -0.19(-0.63, 0.37) | -0.26(-0.63, 0.18) | 0.75(-0.26, 1.42) | 2.58E-08 | 4.47E-08 |
| GP23 | 0.05(-0.47, 0.58) | 0.15(-0.31, 0.68) | 0.17(-0.67, 0.66) | 5.81E-01 | 6.04E-01 |
| GP24 | 0.15(-0.43, 0.70) | 0.36(-0.19, 0.72) | -0.12(-0.99, 0.66) | 8.42E-04 | 1.17E-03 |
| IGP24 | 0.03(-0.38, 0.55) | 0.17(-0.30, 0.54) | -0.09(-0.88, 0.66) | 1.01E-01 | 1.09E-01 |
| IGP25 | 0.37(0.14, 0.65) | 0.44(0.20, 0.66) | -1.29(-1.93, -0.34) | 1.00E-20 | 3.39E-20 |
| IGP26 | 0.06(-0.40, 0.70) | 0.23(-0.33, 0.67) | -0.17(-1.14, 0.41) | 2.98E-04 | 4.31E-04 |
| IGP27 | 0.33(0.03, 0.66) | 0.41(0.08, 0.77) | -1.02(-1.80, -0.30) | 1.00E-20 | 3.39E-20 |
| IGP28 | 0.08(-0.36, 0.48) | 0.13(-0.30, 0.51) | -0.01(-0.96, 0.69) | 4.86E-01 | 5.13E-01 |
| IGP29 | 0.02(-0.25, 0.20) | 0.01(-0.23, 0.21) | 0.17(-0.39, 1.12) | 2.53E-02 | 3.04E-02 |
| IGP30 | -0.19(-0.62, 0.34) | -0.03(-0.48, 0.40) | 0.64(-0.09, 1.52) | 7.89E-12 | 1.58E-11 |
| IGP31 | 0.39(0.21, 0.59) | 0.36(0.21, 0.54) | -1.31(-2.22, 0.09) | 1.00E-20 | 3.39E-20 |
| IGP32 | 0.39(0.07, 0.63) | 0.49(0.18, 0.66) | -0.88(-1.75, 0.08) | 1.00E-20 | 3.39E-20 |
| IGP33 | 0.01(-0.45, 0.61) | -0.13(-0.61, 0.42) | -0.37(-1.13, 0.44) | 3.41E-04 | 4.83E-04 |
| IGP34 | 0.06(-0.39, 0.54) | -0.05(-0.50, 0.49) | -0.49(-1.13, 0.25) | 5.58E-06 | 8.89E-06 |
| IGP35 | -0.18(-0.58, 0.36) | -0.30(-0.60, 0.24) | 0.06(-0.73, 0.95) | 1.03E-01 | 1.10E-01 |
| IGP36 | -0.05(-0.61, 0.50) | 0.07(-0.49, 0.52) | 0.14(-0.52, 1.02) | 4.92E-02 | 5.48E-02 |
| IGP37 | -0.10(-0.57, 0.45) | 0.01(-0.56, 0.48) | 0.10(-0.50, 1.16) | 3.77E-02 | 4.26E-02 |
| IGP38 | -0.06(-0.56, 0.48) | 0.04(-0.55, 0.52) | 0.13(-0.48, 1.17) | 3.77E-02 | 4.26E-02 |
| IGP39 | 0.07(-0.47, 0.55) | 0.16(-0.26, 0.54) | -0.22(-1.08, 0.49) | 6.49E-03 | 8.29E-03 |
| IGP40 | 0.18(-0.34, 0.58) | 0.26(-0.12, 0.57) | -0.09(-1.00, 0.53) | 6.49E-03 | 8.29E-03 |
| IGP41 | -0.07 (-0.69, 0.69) | -0.14 (-0.74, 0.59) | -0.15 (-0.80, 0.45) | 1.80E-05 | 2.81E-05 |
| IGP42 | 0.04(-0.83, 0.64) | 0.02(-0.65, 0.69) | 0.47(-0.19, 0.97) | 4.68E-03 | 6.19E-03 |
| IGP44 | 0.08(-0.40, 0.63) | -0.09(-0.51, 0.53) | -0.46(-1.03, 0.01) | 1.31E-09 | 2.32E-09 |
| IGP43 | 0.19(-0.56, 0.78) | 0.04(-0.59, 0.75) | -0.19(-0.96, 0.52) | 7.78E-03 | 9.63E-03 |
| IGP46 | -0.18(-0.85, 0.38) | -0.13(-0.62, 0.39) | 0.66(-0.27, 1.45) | 6.47E-11 | 1.20E-10 |
| IGP45 | 0.03(-0.64, 0.62) | 0.19(-0.57, 0.79) | 0.07(-0.52, 0.66) | 6.06E-01 | 6.22E-01 |
| IGP47 | 0.30(0.23, 0.38) | 0.28(0.20, 0.37) | -0.26(-1.15, 0.21) | 1.00E-20 | 3.39E-20 |
| IGP48 | -0.11(-0.57, 0.28) | -0.17(-0.63, 0.25) | 0.62(-0.16, 1.83) | 8.84E-14 | 1.97E-13 |
| IGP49 | -0.15(-0.82, 0.34) | -0.19(-0.72, 0.33) | 0.77(-0.16, 1.94) | 4.23E-12 | 8.69E-12 |
| IGP50 | -0.34(-0.60, -0.13) | -0.36(-0.66, -0.19) | 1.80(-0.10, 2.39) | 1.00E-20 | 3.39E-20 |
| IGP51 | -0.10(-0.86, 0.47) | -0.02(-0.59, 0.62) | 0.61(-0.71, 1.28) | 1.62E-04 | 2.42E-04 |
| IGP52 | -0.35(-0.73, 0.13) | -0.34(-0.66, 0.01) | 1.10(-0.13, 1.87) | 6.85E-18 | 1.84E-17 |
| IGP53 | 0.31(0.06, 0.53) | 0.32(0.04, 0.58) | -0.25(-1.35, 0.16) | 1.00E-20 | 3.39E-20 |
| IGP54 | -0.38(-0.65, -0.16) | -0.40(-0.63, -0.17) | 1.61(0.28, 2.37) | 1.00E-20 | 3.39E-20 |
| IGP55 | 0.11(-0.54, 0.77) | 0.08(-0.55, 0.69) | -0.09(-0.93, 0.54) | 3.75E-02 | 4.26E-02 |
| IGP56 | 0.18(-0.31, 0.50) | 0.13(-0.39, 0.40) | 0.08(-0.64, 0.89) | 6.15E-01 | 6.23E-01 |
| IGP57 | -0.10(-0.62, 0.57) | 0.09(-0.64, 0.65) | 0.32(-0.40, 1.04) | 3.28E-02 | 3.88E-02 |
| IGP58 | 0.38(-0.17, 0.85) | 0.36(-0.23, 0.73) | -0.68(-1.35, 0.11) | 2.07E-15 | 5.05E-15 |
| IGP59 | 0.18(-0.33, 0.74) | 0.28(-0.33, 0.67) | -0.19(-1.01, 0.47) | 2.98E-04 | 4.31E-04 |
| IGP60 | 0.40(-0.10, 0.73) | 0.36(-0.09, 0.64) | -0.43(-1.28, 0.39) | 1.03E-10 | 1.87E-10 |
| IGP61 | 0.36(-0.01, 0.67) | 0.30(-0.09, 0.58) | -0.32(-1.05, 0.25) | 8.11E-13 | 1.76E-12 |
| IGP62 | 0.43(0.20, 0.66) | 0.42(0.15, 0.62) | -1.28(-2.03, -0.49) | 1.00E-20 | 3.39E-20 |
| IGP63 | 0.15(-0.42, 0.81) | 0.14(-0.61, 0.66) | -0.14(-0.90, 0.44) | 4.49E-03 | 6.03E-03 |
| IGP64 | 0.37(0.14, 0.67) | 0.37(0.04, 0.63) | -0.90(-2.15, -0.06) | 1.00E-20 | 3.39E-20 |
| IGP65 | 0.35(0.30, 0.39) | 0.34(0.30, 0.38) | -0.53(-1.49, 0.03) | 1.00E-20 | 3.39E-20 |
| IGP66 | -0.35(-0.83, -0.04) | -0.35(-0.75, 0.02) | 1.54(0.74, 2.04) | 1.00E-20 | 3.39E-20 |
| IGP67 | 0.02(-0.73, 0.58) | 0.01(-0.53, 0.64) | 0.15(-0.48, 0.95) | 8.30E-02 | 9.11E-02 |
| IGP68 | -0.27(-0.84, 0.08) | -0.36(-0.75, 0.16) | 1.21(0.22, 2.06) | 1.00E-20 | 3.39E-20 |
| IGP69 | -0.41(-0.62, -0.25) | -0.44(-0.61, -0.24) | 1.70(0.39, 2.34) | 1.00E-20 | 3.39E-20 |
| IGP70 | -0.37(-0.77, -0.08) | -0.37(-0.71, -0.01) | 1.51(0.68, 2.02) | 1.00E-20 | 3.39E-20 |
| IGP71 | -0.35(-0.81, -0.04) | -0.36(-0.73, 0.03) | 1.56(0.76, 2.01) | 1.00E-20 | 3.39E-20 |
| IGP72 | 0.38(0.10, 0.76) | 0.39(0.02, 0.70) | -1.51(-2.01, -0.68) | 1.00E-20 | 3.39E-20 |
| IGP73 | -0.36(-0.72, 0.12) | -0.33(-0.67, -0.02) | 1.09(-0.14, 1.87) | 4.21E-18 | 1.22E-17 |
| IGP74 | -0.41(-0.53, -0.29) | -0.42(-0.52, -0.28) | 1.20(0.19, 2.05) | 1.00E-20 | 3.39E-20 |
| IGP75 | -0.44(-0.63, -0.25) | -0.45(-0.62, -0.23) | 1.72(0.47, 2.36) | 1.00E-20 | 3.39E-20 |
| IGP76 | 0.44(0.29, 0.54) | 0.43(0.27, 0.54) | -1.20(-1.98, -0.49) | 1.00E-20 | 3.39E-20 |
| IGP77 | -0.37(-0.74, 0.08) | -0.35(-0.67, 0.03) | 0.96(-0.12, 1.59) | 4.50E-18 | 1.25E-17 |

The interquartile range (P_25_, P_75_) were given for the GPs and IGPs distribution;

*P*: Kruskal-Wallis H test used for the controls, precancerous and early cancer populations.

Adj. *P：**P*-value was adjusted by Benjamini-Hochberg method.

**Supplementary Table S5**

Comparisons of AUCs between different groups in the discovery and validation populations

|  | ***Z*** | ***P*** |
| --- | --- | --- |
| **Discovery** |  |  |
| Control vs. Precancerosis | 3.162 | 0.002 |
| Precancerosis vs. Early ESCC | 9.282 | <0.001 |
| Control vs. Early ESCC | 6.253 | <0.001 |
| **Validation** |  |  |
| Control vs. Precancerosis | 0.972 | 0.332 |
| Precancerosis vs. Early ESCC | 3.808 | <0.001 |
| Control vs. Early ESCC | 2.933 | 0.004 |

*P*: DeLong’s test used for the comparison of AUCs between different groups.

**Supplementary Table S6**

SNPs associated with ESCC-related IgG GP and IGPs.

|  | SNP | EA | OA | ***EAF*** | ***Beta*** | ***P*** | ***R^2^*** | Symbol | Gene | Feature | Biotype |
| --- | --- | --- | --- | --- | --- | --- | --- | --- | --- | --- | --- |
| GP20 | rs12986368 | T | C | 0.371 | 0.116 | 3.39E-11 | 0.006 | FUT6 | ENSG00000156413 | ENST00000286955 | protein_coding |
| IGP33 | rs17630758 | A | G | 0.152 | -0.138 | 1.17E-09 | 0.005 | SMARCB1 | ENSG00000099956 | ENST00000263121 | protein_coding |
| IGP 33 | rs2309748 | A | T | 0.647 | -0.105 | 3.38E-09 | 0.005 | AFF3 | ENSG00000144218 | ENST00000317233 | protein_coding |
| IGP 33 | rs4821897 | A | G | 0.260 | 0.226 | 5.29E-31 | 0.020 | TAB1 | ENSG00000100324 | ENST00000331454 | protein_coding |
| IGP 33 | rs6570330 | A | G | 0.428 | -0.099 | 6.52E-09 | 0.005 | NA | NA | NA | NA |
| IGP 33 | rs9276726 | T | C | 0.645 | 0.114 | 4.04E-08 | 0.006 | NA | NA | NA | NA |
| IGP 44 | rs1122979 | A | G | 0.112 | -0.208 | 1.48E-14 | 0.009 | ABCF2 | ENSG00000033050 | ENST00000222388 | protein_coding |
| IGP 44 | rs17630758 | A | G | 0.152 | -0.182 | 1.83E-15 | 0.009 | SMARCB1 | ENSG00000099956 | ENST00000263121 | protein_coding |
| IGP 44 | rs3824459 | T | C | 0.015 | -0.438 | 6.02E-09 | 0.006 | NA | NA | NA | NA |
| IGP 44 | rs6583437 | A | G | 0.372 | -0.118 | 1.49E-11 | 0.006 | IKZF1 | ENSG00000185811 | ENST00000331340 | protein_coding |
| IGP 44 | rs7281587 | A | G | 0.247 | -0.144 | 1.13E-13 | 0.008 | RUNX1 | ENSG00000159216 | ENST00000475045 | protein_coding |
| IGP 44 | rs909674 | A | C | 0.734 | -0.194 | 9.10E-24 | 0.015 | MGAT3 | ENSG00000128268 | ENST00000341184 | protein_coding |
| IGP 58 | rs10149325 | A | G | 0.581 | -0.212 | 8.38E-16 | 0.022 | NA | NA | NA | NA |
| IGP 58 | rs11651000 | A | G | 0.150 | -0.165 | 2.66E-12 | 0.007 | NA | NA | NA | NA |
| IGP 58 | rs11652097 | T | C | 0.389 | -0.099 | 1.05E-08 | 0.005 | NA | NA | NA | NA |
| IGP 58 | rs11847263 | T | G | 0.647 | 0.282 | 4.85E-58 | 0.036 | CTD-2509G16.5 | ENSG00000258760 | ENST00000553754 | lincRNA |
| IGP 58 | rs7216389 | T | C | 0.487 | 0.137 | 1.17E-15 | 0.009 | GSDMB | ENSG00000073605 | ENST00000309481 | protein_coding |
| IGP 58 | rs7789913 | T | C | 0.395 | 0.171 | 1.18E-23 | 0.014 | NA | NA | NA | NA |
| IGP 58 | rs7812088 | A | G | 0.119 | 0.208 | 5.81E-16 | 0.009 | NA | NA | NA | NA |
| IGP 75 | rs10813951 | A | G | 0.732 | 0.181 | 1.80E-21 | 0.013 | B4GALT1 | ENSG00000086062 | ENST00000379731 | protein_coding |
| IGP 75 | rs10903118 | T | C | 0.484 | 0.115 | 6.97E-12 | 0.007 | RUNX3 | ENSG00000020633 | ENST00000338888 | protein_coding |
| IGP 75 | rs11249231 | A | G | 0.101 | -0.171 | 6.26E-10 | 0.005 | NA | NA | NA | NA |
| IGP 75 | rs17630758 | A | G | 0.152 | 0.237 | 2.81E-25 | 0.014 | SMARCB1 | ENSG00000099956 | ENST00000263121 | protein_coding |
| IGP 75 | rs6583437 | A | G | 0.372 | 0.143 | 1.90E-16 | 0.010 | IKZF1 | ENSG00000185811 | ENST00000331340 | protein_coding |
| IGP 75 | rs7281587 | A | G | 0.248 | 0.126 | 6.37E-11 | 0.006 | RUNX1 | ENSG00000159216 | ENST00000475045 | protein_coding |
| IGP 75 | rs8137426 | T | G | 0.255 | -0.156 | 5.79E-15 | 0.009 | NA | NA | NA | NA |
| IGP 75 | rs9385856 | T | C | 0.574 | -0.102 | 1.97E-09 | 0.005 | NA | NA | NA | NA |

SNP: variant with the strongest association in the locus; EA: allele for which effect estimate is reported; OA: other allele; EAF: frequency of the effect allele; Beta: effect estimate for the SNP and glycan with the strongest association in the locus in the discovery; P: P value for the discovery effect estimate; R2: sample size weighted average of imputation quality for the SNP with the strongest association in the locus.
