# Supplementary figures and images for: Identification and validation of IgG N-glycosylation biomarkers of esophageal carcinoma
Source: Front Immunol. 2023 Mar 14;14:981861. doi: 10.3389/fimmu.2023.981861 (PMC10043232; doi:10.3389/fimmu.2023.981861)

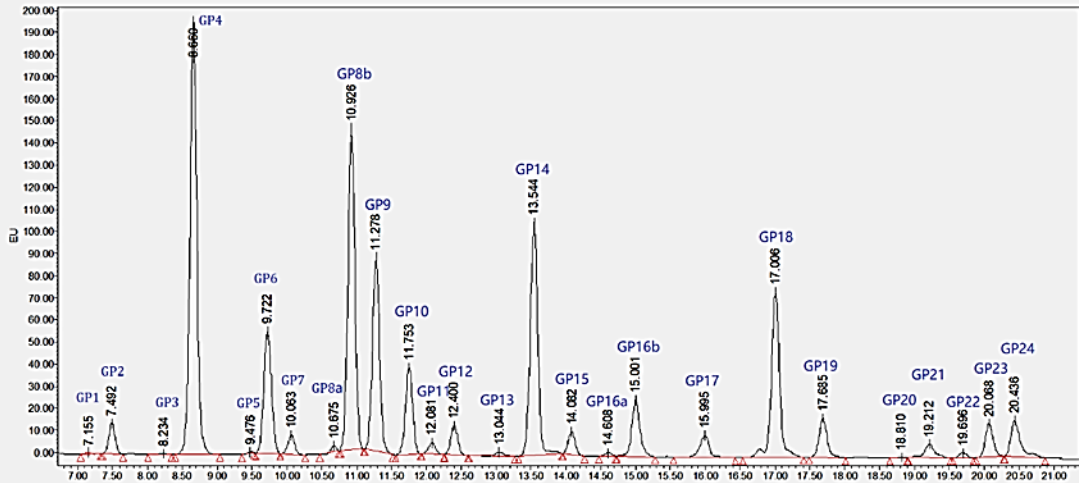

**A**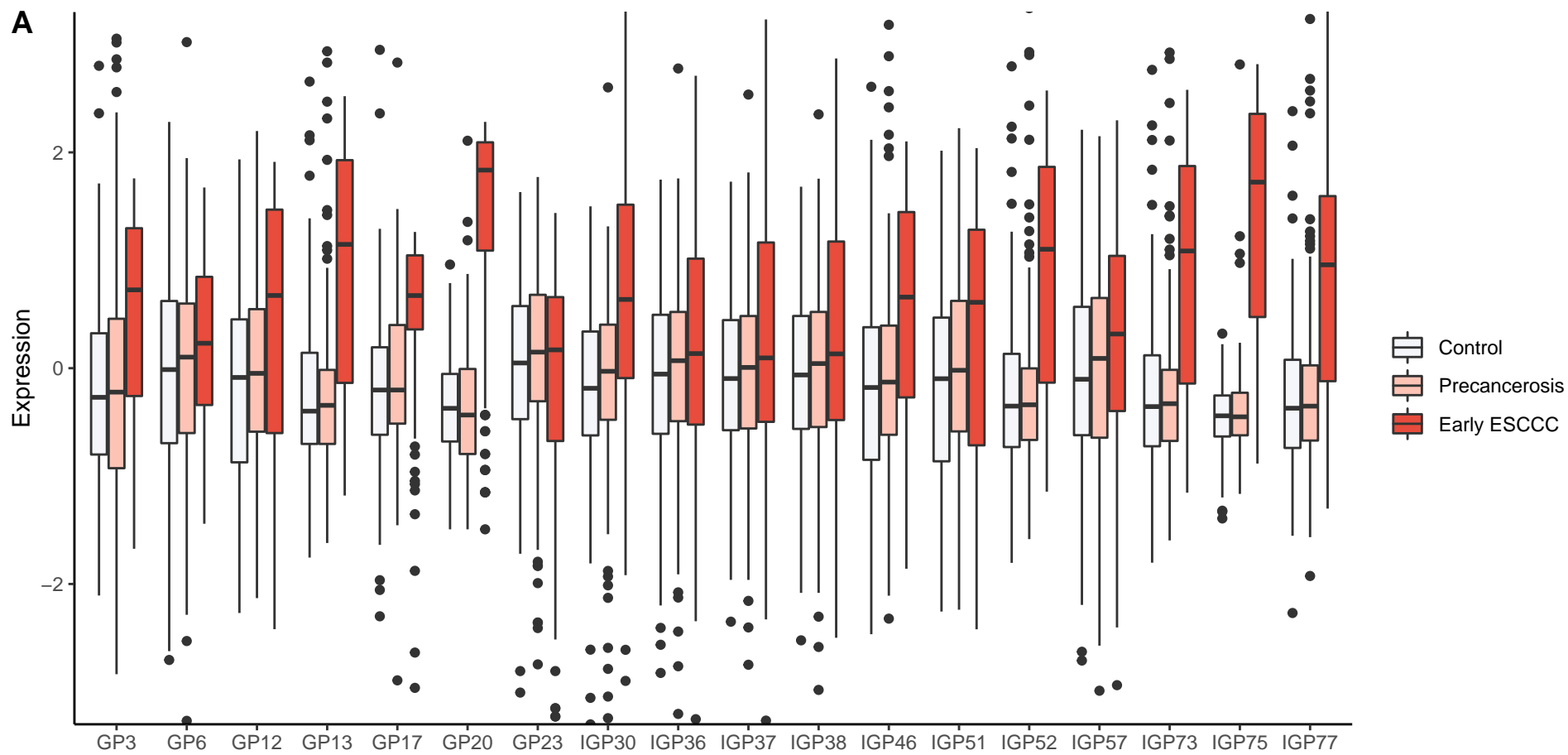**B**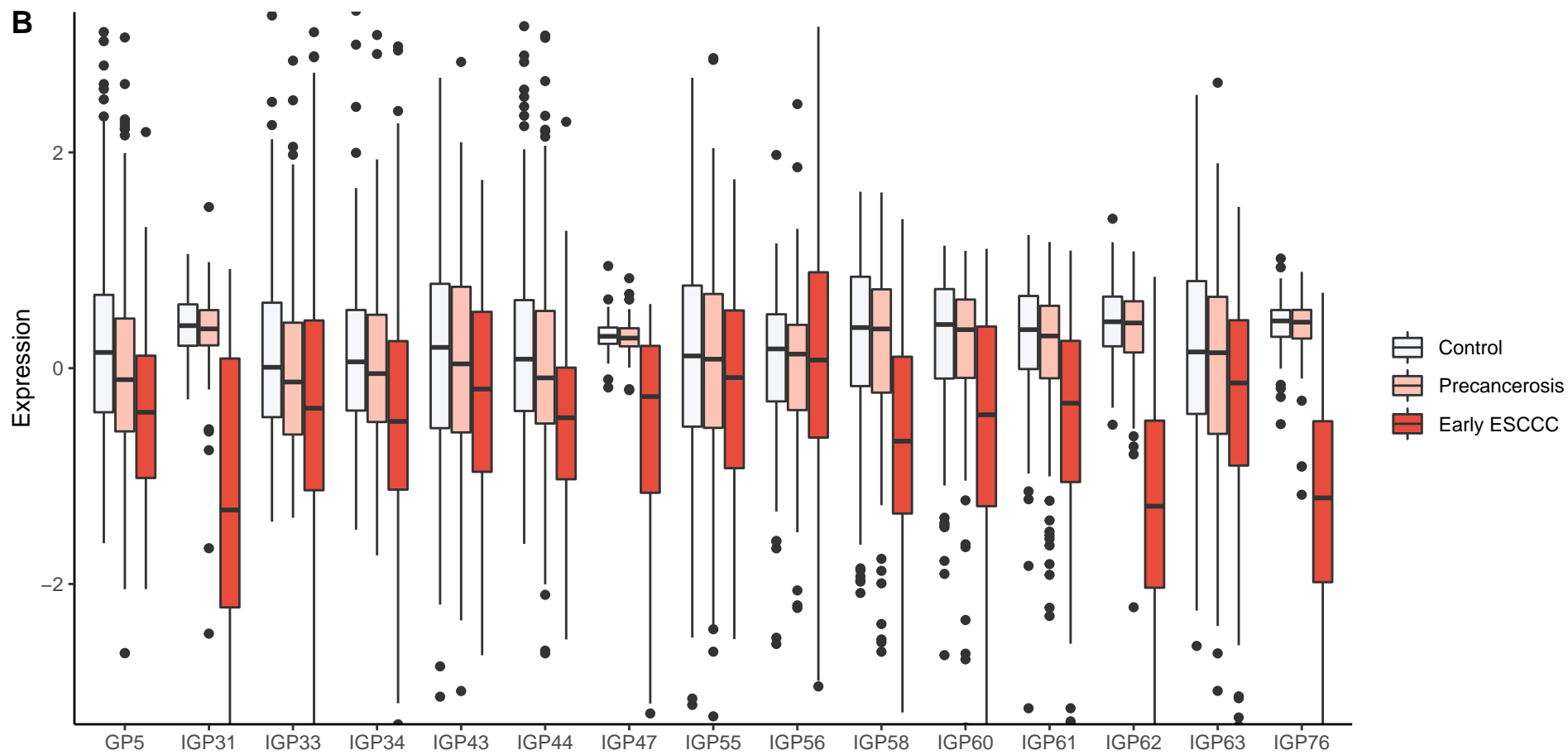

# KEGG

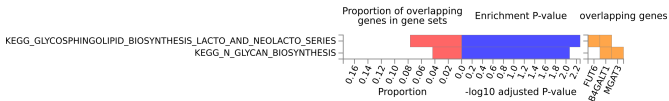

# GO

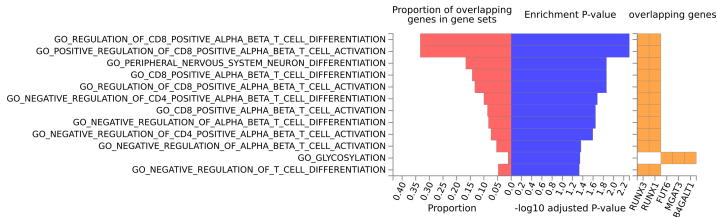

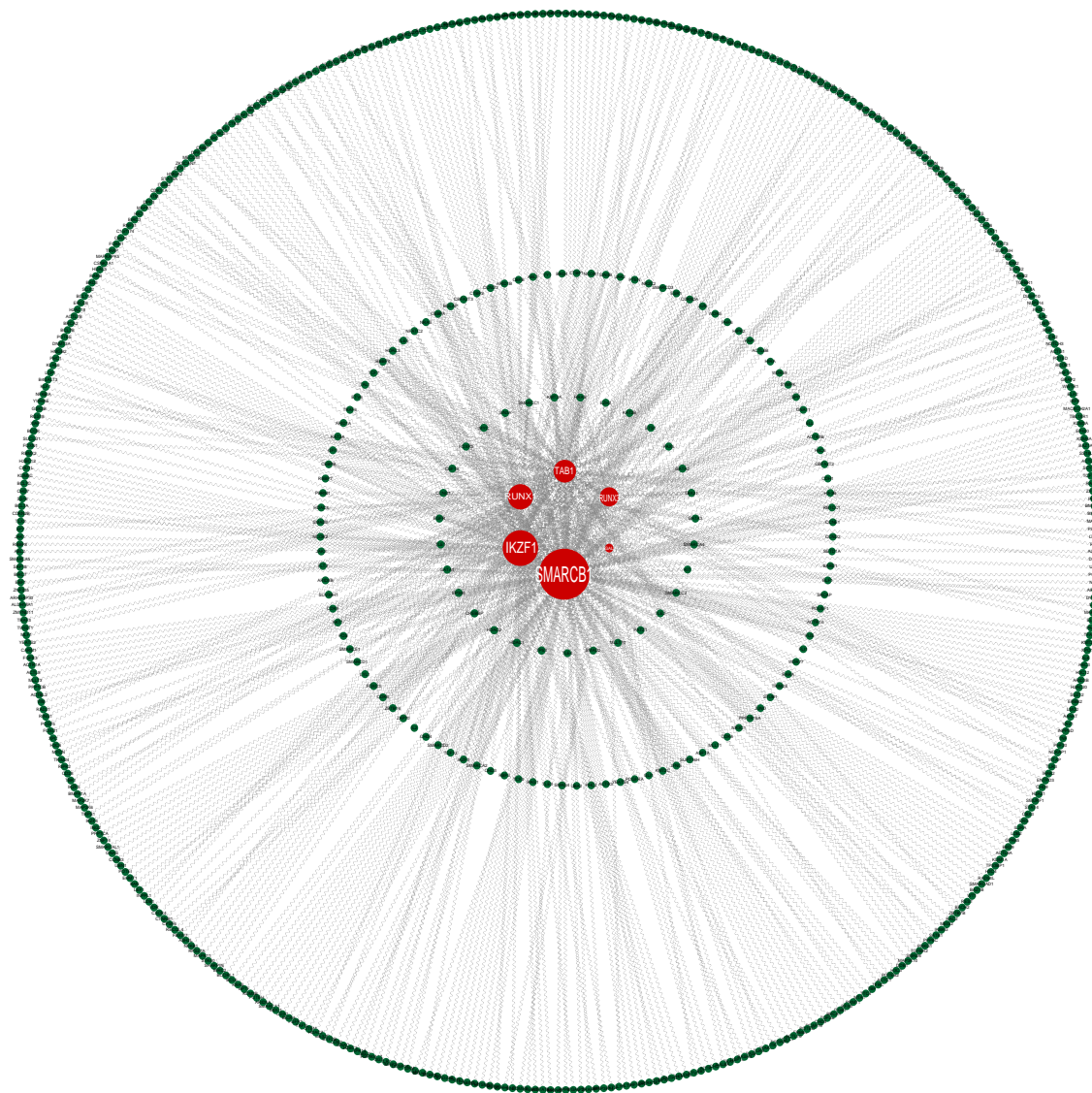

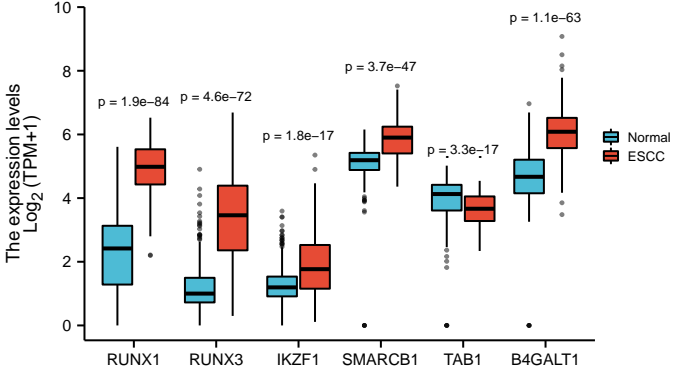

Supplement: Supplementary Figure 1 — UPLC analysis of the IgG glycome. IgG glycome was separated into 24 chromatographic peaks by hydrophilic interaction chromatography. [file Image_1.pdf]
